# Werner syndrome with refractory cystoid macular edema and immunohistochemical analysis of WRN proteins in human retinas

**DOI:** 10.1186/1471-2415-14-31

**Published:** 2014-03-12

**Authors:** Toshiyuki Oshitari, Masayasu Kitahashi, Satoshi Mizuno, Takayuki Baba, Mariko Kubota-Taniai, Minoru Takemoto, Koutaro Yokote, Shuichi Yamamoto, Sayon Roy

**Affiliations:** 1 Department of Ophthalmology and Visual Science, Chiba University Graduate School of Medicine, Inohana 1-8-1, Chuo-ku, Chiba 260-8670, Chiba, Japan; 2 Departments of Medicine and Ophthalmology, Boston University School of Medicine, 715 Albany Street, Boston, MA 02118, USA; 3 Department of Medicine, Division of Diabetes, Metabolism and Endocrinology, Chiba University Hospital, Inohana 1-8-1, Chuo-ku, Chiba 260-8670, Chiba, Japan

**Keywords:** Refractory cystoid macular edema, WRN, Retina, Müller cell, Immunohistochemistry

## Abstract

**Background:**

To present our findings in a case of Werner syndrome with refractory cystoid macular edema (CME) and to determine the expression and the distribution of WRN proteins in human retinas.

**Case presentation:**

A 35-year-old man with Werner syndrome who developed CME after YAG laser treatment was studied. Optical coherence tomographic (OCT) scans were used to examine the CME in the right eye. The patient received topical eye drops (0.1% bromfenac sodium hydrate twice daily and 1% dorzolamide hydrochloride thrice daily), sub-Tenon triamcinolone injection thrice, intravitreal bevacizumab injection twice, and pars plana vitrectomy of the right eye. Genetic analyses were performed to diagnose the disease. To examine the expression and distribution of WRN proteins in the retinas, immunohistochemistry for WRN proteins was performed in human retinas. The CME in the right eye was not improved by any of the treatments. During the follow-up period, CME developed in the left eye. Genetic analyses detected compound heterozygosity, Mut4 and Mut11, in the *WRN* gene and the individual was diagnosed with Werner syndrome. Immunohistochemical analysis of WRN proteins expression in human retinas showed that WRN proteins were expressed in the parts of the Müller cells in the inner nuclear layer and outer nuclear layer.

**Conclusion:**

Patients with Werner syndrome can develop severe CME after laser treatment. A pathological link may exist between mutations in the *WRN* gene and the development of CME in patients with Werner syndrome.

## Background

Werner syndrome is one of the genetic progeria disorders with autosomal recessive inheritance, and it is caused by mutations of the Werner syndrome (*WRN*) gene [1-3]. The *WRN* gene is found on chromosome 8p12 which is located at a RecQ type DNA/RNA helicase [1-3]. The wild type WRN protein is associated with replication, repair, recombination, and transcription of DNA [1-3]. The results of an earlier study indicated that WRN proteins were present in neuronal and glial cells in the brain [4]. However, there has been no report of their expression and distribution in adult human retinas.

Werner syndrome is considered to be a model of human aging and is characterized by baldness, cataracts in the twenties, atrophy of the skin, type II diabetes mellitus usually in the thirties, arteriosclerosis in the forties, and increased incidences of malignant cancers in the mid-forties [1-3,5]. Only 1400 individuals have been diagnosed with Werner syndrome in the world and 75% of them are Japanese [5].

The initial finding of patients with Werner syndrome has been juvenile cataracts found during ophthalmological examinations [6]. However, the incidence of eye diseases has been low compared to skin atrophy and sclerosis, prominent characteristics of patients with Werner syndrome [5].

We report a case of Werner syndrome accompanied by refractory cystoid macular edema (CME). In addition, we determined the expression and the distribution of the WRN proteins in adult human retinas by immunohistochemistry.

## Case presentation

A 35-year-old man underwent bilateral cataract surgeries at 25-years-of-age. At the 32 years, he underwent Neodymiun-YAG laser posterior capsulotomy in the right eye because his right visual acuity had decreased to 0.7. Immediately after the YAG laser treatment, CME developed in the right eye and he was referred to Chiba University Hospital.

**Figure 1:**
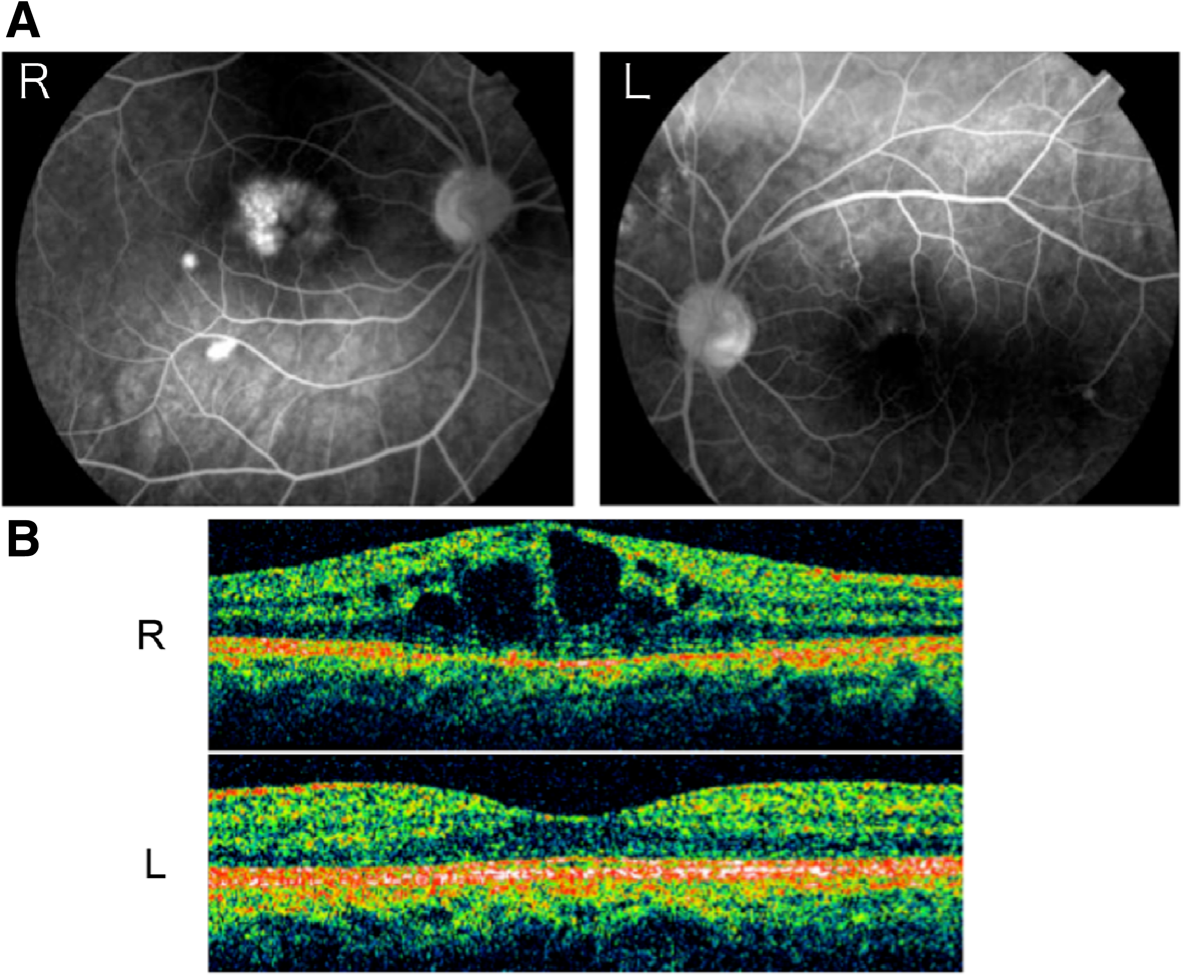
**Initial findings of retinal abnormalities in patient with Werner syndrome.** Fluorescein angiographic **(FAG; A)** and optical coherence tomographic **(OCT; B)** findings. The patient’s visual acuity was 0.5 OD. CME was detected in the right eye in both FAG and OCT images. FAG showed high fluorescent signals near the lower arcade of the right eye indicating damage of the retinal pigment epithelium **(A)**.

Our examination showed that his decimal visual acuities were 0.5 OD and 1.0 OS. The intraocular pressures were within normal limits. The anterior segments were normal and both eyes were pseudophakic but fluorescein angiography (FAG) and optical coherence tomographic (OCT) scans showed CME in the right eye (Figure 1).

At 27-years-of-age, he underwent bipolar hip arthroplasty for bilateral idiopathic osteonecrosis of the femoral heads. Around this period, the patient noticed a loss of hair and hallux valgus. At the age of 33 years, he underwent skin grafting for a skin ulcer on his right leg, and at 35 years, bilateral calcification of the achilles tendon was detected from X-ray images. Atrophy of the skin in both legs was also found. He had no family history of Werner syndrome.

According to the cardinal signs and symptoms of the diagnostic criteria for Werner syndrome on the International Registry website, the patient had bilateral cataract, characteristic dermatological pathology including ulceration, atrophic skin, and thinning of the scalp hair. Additional examinations revealed that the patient had hypogonadism, osteoporosis, soft tissue calcification, and hoarse voice that are further signs of Werner syndrome. These characteristics in our patient met the diagnostic criteria of "Probable Werner syndrome" on the International Registry of Werner syndrome website ( http://www.wernersyndrome.org/registry/diagnostic.html).

Our patient underwent genetic analysis in the Department of Medicine of Chiba University Hospital and was diagnosed with Werner syndrome. This was based on *WRN* gene, also called RecQL2 or RecQ3, DNA mutation analysis, which indicated that the patient carried the Mut4 and Mut11 mutations (compound heterozygote) in the *WRN* gene of the RecQ helicase gene family. The Werner syndrome protein, WRN, is one of five conserved members of the RecQ helicase family associated with premature aging [1-3].

**Figure 2:**
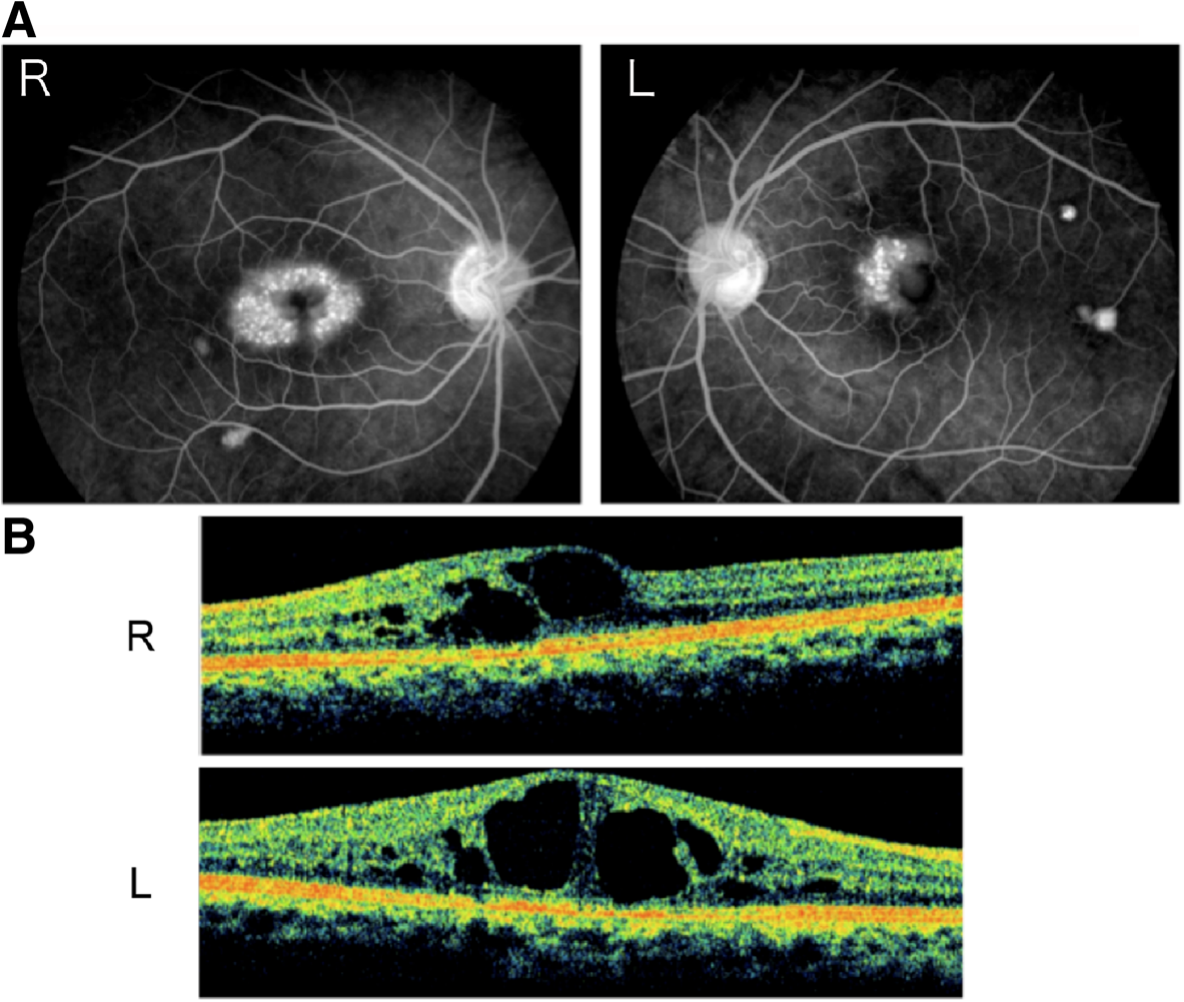
**Retinal abnormalities detected by FAG (A) and OCT (B) 1 year after vitrectomy.** The patient’s visual acuities were 1.0 OU. CME developed in both eyes **(A, B)**. FAG shows new window defects near the upper arcade of the left eye **(A)**.

We first considered a diagnosis of Irvine-Gass syndrome after Neodymium-YAG laser posterior capsulotomy [7], and used topical 0.1% bromfenac sodium hydrate and 1% dorzolamide hydrochloride. In addition, sub-tenon administration of 20 mg triamcinolone acetonide (STTA) was given, and the CME slightly improved and his visual acuity improved to 1.0 OD. However during the followed-up period, a recurrence of the CME was observed. Although he underwent three STTA and two intravitreal bevacizumab (IVB) injections, the CME could not be improved. He finally underwent pars plana vitrectomy to create a posterior vitreous detachment but the CME did not improve. His right visual acuity was subsequently 1.0 OD but he suffered from metamorphopsia. During the follow-up period, CME developed in his left eye (Figure 2) despite receiving 1% dorzolamide hydrochloride eye drops and good visual acuity without metamorphopsia.

### Immunohistochemistry

Five normal eyes (64 ± 12 years) were obtained from the National Disease Research Interchange (NDRI, Philadelphia, PA). The human donor eyes from the NDRI were delivered within 48 hours postmortem on ice. The donor information provided from NDRI indicated that the eyes were normal with no ocular eye diseases and no diabetes mellitus. Upon receiving the eyes, the retinas were immediately isolated followed by fixation in 4% paraformaldehyde for immunohistochemistry. The retinas were cryosectioned and stored at -20°C.

Epiretinal membranes that were surgically removed from patients with proliferative diabetic retinopathy were fixed in 4% paraformaldehyde and used as positive controls for WRN immunohistochemistry. The epiretinal membranes were cryosectioned and stored at -20°C.

After blocking with 3% bovine serum albumin and 5% goat serum in 0.1 M phosphate buffer saline (PBS), sections were incubated with rabbit anti-WRN antibody (H-300; Santa Cruz Biotechnology, Santa Cruz, CA; sc-5629) at 4°C overnight [8-13]. After washing with 0.1 M PBS, the retinal and epiretinal membrane sections were incubated with fluorescein isothiocyanate (FITC)-conjugated anti-rabbit IgG for 1 h at room temperature. Next, the retinal sections were washed with 0.1 M PBS and incubated with mouse anti-vimentin, glial fibrillary acidic protein (GFAP), rhodopsin or cone arrestin antibodies for 3 h at room temperature. Finally after washing with 0.1 M PBS, the sections were labeled with rhodamine B isothiocyanate (RITC)-conjugated anti-mouse antibody for 1 h at room temperature. The retinal and epiretinal membrane sections were co-stained with 4, 6-diamidino-2-phenyl indole (DAPI) to label nuclei. The specificity of the antibodies was confirmed by omitting the primary antibodies. After mounting, the sections were immediately observed under a fluorescence microscope. This research protocol was approved by the Institutional Review Board of the hospital. All procedures were performed according to the provisions of the Declaration of Helsinki.

**Figure 3:**
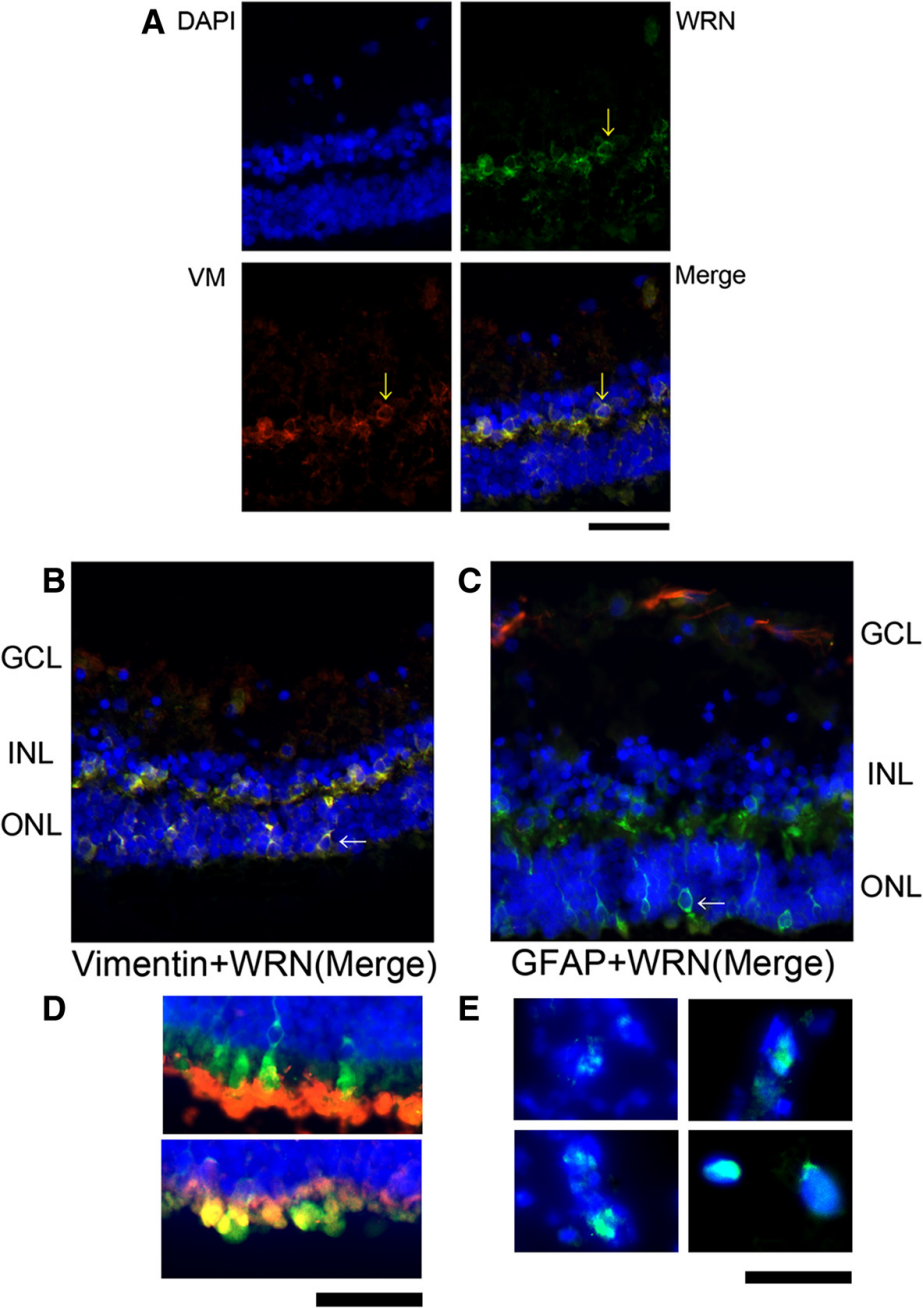
**Expression and localization of WRN proteins in human retinas.** WRN protein signals (green) were detected in the inner nuclear layer (INL; yellow arrows) **(A)** and the outer nuclear layer (ONL; white arrow) **(B, C)**. The WRN protein signals co-existed with vimentin signals (red; yellow arrows) **(A)** but not with GFAP signals **(red; C)**. These results indicate that the WRN proteins are expressed in the cytosol of Müller cells in human retinas. The upper picture of Panel **D** shows the rhodopsin signals (red) coexisted with WRN signals (green) in the ONL **(D)**. The lower picture of Panel **D** shows the cone arrestin signals (red) coexisted with WRN signals (green) in the ONL **(D)**. Panel E shows expression and localization of WRN proteins in epiretinal membrane of another patient with proliferative diabetic retinopathy. WRN protein signals (green) were identified in the nucleus **(E)**. Blue signals showed DAPI-stained nuclei. Blue signals showed DAPI-stained nuclei. GCL; ganglion cell layer, INL; inner nuclear layer, ONL; outer nuclear layer, VM; vimentin, GFAP; glial fibrillary acidic protein. Bars = 20 μm.

In adult human retinas, the WRN proteins are expressed mainly in the Müller cells of the inner nuclear layer (INL), and partially in the outer nuclear layer (ONL) and in the outer plexiform layer (Figure 3A-C). WRN positive cells had vimentin-positive signals, but not GFAP-positive signals, and the WRN-positive cells in the ONL co-existed with vimentin-positive signals (Figure 3A-C). These findings indicated that the WRN proteins were expressed in Müller cells in the INL or the ONL but not expressed in astrocytes [14,15]. In addition, WRN proteins were partially expressed in both rods and cones of the ONL (Figure 3D). The localization of WRN protein expression in Müller cells was not in the nucleus but in the cytosol especially surrounding the nucleus (Figure 3A-C). On the other hand, WRN expression in the epiretinal membrane was identified in the nucleus (Figure 3E). These results suggested that the WRN proteins expressed in Müller cells may have other roles than that of a DNA helicase in the nucleus.

## Conclusions

Earlier, Jonas et al. reported that three of eighteen eyes with Werner syndrome that underwent cataract surgery developed postoperative CME [16]. Kocabora et al. reported on a sister and a brother with Werner syndrome who underwent bilateral cataract surgeries, and one of the four eyes developed postoperative CME but it was easily treated with topical indomethacin [17]. CME is also frequently present in patients with retinitis pigmentosa and Usher syndrome [18,19]. However, our patient had normal retinal function in both eyes.

Vitreous traction was also not the cause of the CME in our case because vitrectomy did not resolve the CME. In addition, the CME was not due to inflammation because it did not improve after IVB and STTA. Although the CME in the right eye may be related to the Irvine-Gass syndrome, CME also developed in the left eye. Thus, the pathogenesis of CME was most likely associated with the mutations in the *WRN* gene.

The *WRN* gene, also called the *RecQL2* or *RecQ3* gene, is identified as a RecQ type DNA/RNA helicase [20]. There are five members of the RecQ helicases; WRN and RecQ4 are members of the RecQ helicases associated with premature aging and cancer predisposition [1-3,5]. More than 30 *WRN* mutations have been identified in patients with Werner syndrome [1-3,5]. Some Werner syndrome patients develop type II diabetes mellitus [1-3,5], which may be related to abnormal WRN expression in the pancreas [21].

Gee et al. demonstrated that the WRN proteins were expressed in neurons and glial cells of the brain [4]. In addition, they identified WRN protein expression in the very distal regions of neuritic processes of brain primary neurons in vitro [4]. Our results showed that the WRN proteins were expressed in the cytosol of Müller cells of normal retinas from elderly people. It is possible that WRN proteins may have other functions than that of DNA helicase or exonuclease in the cytosol. For example, WRN proteins may be involved in the regulation of apoptosis because they can interact with p53 and activate p53-dependent transcription of p21/Waf1 [1,22]. The results of earlier studies indicated that pathological changes, such as swelling and death of Müller cells, were closely related to the onset of CME [23,24]. Laeffler et al. suggested that the pathologic changes of dominantly inherited CME affected mainly Müller cells [24]. The absence of WRN protein expression in Müller cells in patients with Werner syndrome may cause the development of the CME associated with Müller cell dysfunction [25].

Taken together, our results suggest a pathophysiological link between WRN protein expression in Müller cells and the development of refractory CME associated with Werner syndrome.

In conclusion, we report a case of refractory CME that developed in a patient with Werner syndrome. The expression of WRN proteins in Müller cells of adult human retinas indicates that WRN gene is active in Müller cells, and therefore a pathophysiological link may exist between the mutation in the *WRN* gene and the development of CME in patients with Werner syndrome. Further studies are needed to demonstrate the precise role of WRN proteins expression in Müller cells and their association with the development of CME.

## Consent

Written informed consent was obtained from the patient for publication of this Case report and any accompanying images. A copy of the written consent is available for review by the Editor of this journal.

## Abbreviations

CME: Cystoid macular edema; FAG: Fluorescein angiography; OCT: Optical coherence tomography; NDRI: National Disease Research Interchange; PBS: Phosphate buffer saline; FITC: Fluorescein isothiocyanate (FITC); GFAP: Glial fibrillary acidic protein; RITC: Rhodamine B isothiocyanate; DAPI: 4,6-diamidino-2-phenyl indole; STTA: Sub-tenon administration of triamcinolone acetonide; IVB: Intravitreal bevacizumab; INL: Inner nuclear layer; ONL: Outer nuclear layer.

## Competing interests

The authors declare that they have no financial conflict of interests.

## Authors’ contributions

TO, KM, SR, SY designed of the study. TO, SM, TB, MK conducted of the study. TO, SM, TB, MK analyzed the data. MT, KY performed the genetic analysis of the patient. TO, SM, SR, SY wrote the manuscript. SR, SY edited the manuscript. All authors read and approved the final manuscript.

## Pre-publication history

The pre-publication history for this paper can be accessed here:

http://www.biomedcentral.com/1471-2415/14/31/prepub
